# Evaluating Water, Sanitation and Hygiene (WASH) Services in Healthcare Facilities: A Comparative Cross-Sectional Study Using the WHO Water and Sanitation for Health Facility Improvement Tool (WASH FIT)

**DOI:** 10.7759/cureus.114790

**Published:** 2026-08-19

**Authors:** Hamza H Cheema, Muhammad Suleman, Usman Zafar, Farrukh Ansar, Hassan Shafqat, Mazahir Hussain, Muhammad Awon Raza, Yasif Abbas, Muhammad Imran

**Affiliations:** 1 Environmental Sciences, National University of Sciences and Technology, Islamabad, PAK; 2 Public Health, Alkhidmat Raazi Hospital, Rawalpindi, PAK; 3 Internal Medicine, Alkhidmat Raazi Hospital, Rawalpindi, PAK; 4 General Practice, Alkhidmat Raazi Hospital, Rawalpindi, PAK; 5 General Surgery, Alkhidmat Raazi Hospital, Rawalpindi, PAK

**Keywords:** healthcare facilities, health systems strengthening, infection prevention and control, wash fit, water sanitation and hygiene (wash)

## Abstract

Background

Water, sanitation and hygiene (WASH) services are foundational to safe health care delivery and infection prevention, yet facility-level evidence from Pakistan remains limited. This study assessed and compared WASH services at two hospitals using the World Health Organization’s Water and Sanitation for Health Facility Improvement Tool (WASH FIT).

Methods

A comparative cross-sectional assessment was conducted in December 2025 at two hospitals in Rawalpindi (Hospital A) and Islamabad (Hospital B). Two teams of assessors independently evaluated both facilities through structured observation and record review. Indicators were scored on the standard WASH FIT scale across seven domains, and service levels assigned using JMP core-indicator criteria. To ensure comparison against an identical indicator set, five indicators that did not apply equally to both facilities owing to service configuration or cross-referencing within another domain were excluded, yielding an analytical set of 97 indicators scored in both facilities.

Results

Hospital A scored 147 of 194 points (75.8%) and Hospital B 127 of 194 (65.5%). Hospital A met basic service criteria in four of five domains with defined JMP ladders, compared with two of five in Hospital B. Among these five domains, hand hygiene was strongest, 10/10 (100%) and 9/10 (90%), and sanitation weakest, 15/26 (57.7%) and 12/26 (46.2%). The largest difference between facilities was in management and workforce, 16/24 (66.7%) versus 7/24 (29.2%). Both scored higher on essential than advanced indicators: 76/82 (92.7%) versus 50/82 (61.0%); 67/82 (81.7%) versus 41/82 (50.0%). Sixteen indicators were unmet in both facilities, concentrated in wastewater management, laundry infrastructure, and institutional planning.

Conclusion

Both hospitals demonstrated moderate WASH performance, with the largest difference observed in management and workforce rather than infrastructure. Strengthening governance and workforce capacity alongside infrastructure may improve WASH performance in resource-limited healthcare facilities.

## Introduction

Safe water, sanitation, and hygiene (WASH) services are now recognized as foundational to quality healthcare delivery and health system resilience, yet remain critically inadequate in many settings [1]. The WHO/UNICEF Joint Monitoring Programme (JMP) estimates that one in four healthcare facilities globally lacks basic water services, and nearly half lack basic sanitation, with the burden disproportionately concentrated in low- and middle-income countries (LMICs) [2]. These systemic deficiencies undermine infection prevention and control (IPC), compromise patient safety, and impede progress toward universal health coverage.

The relationship between WASH and clinical outcomes is strong [3]. Inadequate WASH infrastructure facilitates the transmission of healthcare-associated infections (HAIs), which remain a major cause of morbidity and mortality worldwide [4]. Evidence indicates that strengthening WASH services, particularly hand hygiene, environmental cleaning, and healthcare waste management, is essential for preventing healthcare-associated infections, improving patient safety, and enhancing the quality of care [5]. Despite this, WASH services in healthcare facilities are often neglected in health system planning, particularly in resource-constrained environments [6].

To address these gaps, the WHO and UNICEF introduced the Water and Sanitation for Health Facility Improvement Tool (WASH FIT), a facility-level, risk-based framework designed to enable continuous assessment and incremental improvement of WASH services [7]. WASH FIT adopts a comprehensive systems approach, encompassing multiple domains including water supply, sanitation, healthcare waste, hand hygiene, environmental cleaning, energy, and governance structures [7].

In Pakistan, however, the evidence base on WASH performance in healthcare facilities remains limited and fragmented [8]. National assessments highlight significant deficiencies in infrastructure, governance, and maintenance systems, with persistent gaps in sanitation, waste management, and institutional capacity [9]. These shortcomings not only compromise quality of care but also pose substantial risks to healthcare workers and vulnerable patient populations [10]. Despite increasing global attention to WASH in healthcare facilities, evidence from low- and middle-income countries indicates that implementation of standardized WASH improvement frameworks is often incomplete, with limited use of continuous quality improvement cycles and routine follow-up assessments [11].

Given these challenges, there is a critical need for robust, facility-level evaluations to inform evidence-based planning and policy. This study therefore addresses a primary research question through a descriptive, facility-level comparison: how do two hospitals within the same network compare in WASH service performance across the seven WASH FIT domains, and where do their principal strengths and systemic gaps lie? To answer this, we undertake a comparative assessment using the WASH FIT framework, with attention to domain-specific performance, institutional strengths, and shared deficits. This is a descriptive comparison and does not involve inferential or hypothesis-based testing. By generating context-specific evidence, the study aims to support targeted interventions and contribute to strengthening WASH systems in healthcare facilities within resource-limited settings.

## Materials and methods

Study design and setting

A comparative, facility-based cross-sectional assessment was conducted in December 2025 at two hospitals within the Alkhidmat Raazi Hospital network: Alkhidmat Raazi Hospital, Rawalpindi (designated Hospital A) and Alkhidmat Raazi Hospital, Islamabad (Hospital B). Both hospitals provide emergency, inpatient, outpatient, surgical, medical, and obstetric services. Both operate sewered sanitation systems connected to the municipal network and dispose of health care waste through contracted off-site treatment.

Assessment tool

Water, sanitation, and hygiene services (WASH) were assessed using the WHO/UNICEF Water and Sanitation for Health Facility Improvement Tool, second edition, applied via the accompanying WASH FIT Excel workbook [7]. WASH FIT is a risk-based, facility-level framework that evaluates WASH services across seven domains: water; sanitation; health care waste; hand hygiene; environmental cleaning; energy and environment; and management and workforce. Indicators within each domain are classified as essential or advanced according to priority, and are separately identified where they contribute to the WHO/UNICEF JMP core monitoring set or address climate resilience.

Data collection

Assessments were conducted by two teams of assessors drawn from the authors, who were briefed together on the WASH FIT framework and indicator definitions before data collection to promote consistent interpretation and scoring. Each team visited both facilities on separate dates, so that every facility was assessed independently on two occasions. This duplicate assessment was undertaken as a verification procedure to reduce single-observer error. Agreement between the two teams' independent scores, calculated across all 97 indicators in both facilities prior to consensus reconciliation, was high (Cohen's κ = 0.94, 95% CI 0.90-0.98). Data were collected through direct structured observation of infrastructure and review of facility records; no staff was individually observed, interviewed, or surveyed, and no patient-level data were collected at any stage.

The two teams' scoring sheets were subsequently compared indicator by indicator. Where scores differed, the discrepancy was reviewed against the supporting observational and documentary evidence and a consensus score agreed by both teams, with final adjudication by one author (HHC), who is not affiliated with either participating facility. Only consensus scores were entered into the WASH FIT assessment workbook and used in the analysis.

Scoring and analytical set

Each indicator was scored on the standard WASH FIT three-point scale: 0 (does not meet the standard), 1 (partially meets the standard) and 2 (fully meets the standard). Domain and overall scores were calculated as the achieved score divided by the maximum attainable score, defined as twice the number of indicators assessed, and expressed as a percentage.

Service levels were assigned using the JMP core-indicator criteria embedded in the WASH FIT. A domain is classified as basic, limited, or no service according to performance on specified core indicators rather than on its aggregate percentage score; the two measures are therefore reported separately and are not interchangeable. JMP service ladders are defined for the water, sanitation, health care waste, hand hygiene, and environmental cleaning domains, and are not defined for energy and environment or for management and workforce.

To ensure that the two facilities were compared against an identical indicator set, indicators were excluded from the analysis where they did not apply equally to both hospitals. Both facilities operate sewered sanitation, so where WASH FIT provides parallel indicators for sewered and non-sewered systems, only the sewered pathway was scored. Indicators restricted to primary-level facilities, and entries that cross-reference an indicator already scored within another domain, were likewise excluded to avoid double counting. Five indicators were excluded on these grounds: one water indicator applicable only to primary-level facilities, one hand hygiene entry cross-referencing a sanitation indicator, and three sanitation indicators applying to non-sewered systems. The resulting analytical set comprised 97 indicators, all of which were scored in both facilities, giving complete data for every comparison reported.

Analysis

Analysis was descriptive. Scores were summarized by domain and overall for each facility, and differences between facilities were expressed in percentage points. Performance was additionally disaggregated by indicator classification; essential, advanced, JMP core set, and climate resilience to characterize the profile of each facility independently of domain structure. Indicator-level concordance between the two hospitals was calculated as the proportion of the analytical set receiving an identical score in both, and indicators scoring zero in both facilities were tabulated to identify shared rather than facility-specific deficits. No inferential statistical testing was performed. With two facilities assessed in full, there is no sampling distribution against which to test, and all comparisons reported are descriptive. As both facilities were assessed in full rather than sampled, measures of sampling variability such as confidence intervals are not applicable, and differences are reported as absolute percentage points.

Sensitivity analysis

Three health care waste indicators in the analytical set, on-site waste treatment technology, energy or fuel supply for on-site treatment, and dedicated ash pits for incineration residues, apply only to facilities operating on-site waste treatment. Both hospitals dispose of waste through contracted off-site treatment and scored the maximum on the corresponding off-site indicator. A pre-specified sensitivity analysis was therefore conducted in which these three indicators were treated as not applicable and removed from both the numerator and the denominator in both facilities, to determine whether the comparative findings were dependent on their inclusion.

Ethical considerations

Ethical approval for the study was granted by the Institutional Review Board of Alkhidmat Raazi Hospital, Rawalpindi (reference number: IRB/A/07/25). Written administrative permission was obtained from the management of each facility before assessment. The study involved no human participants.

## Results

Overall performance

**Figure 1:**
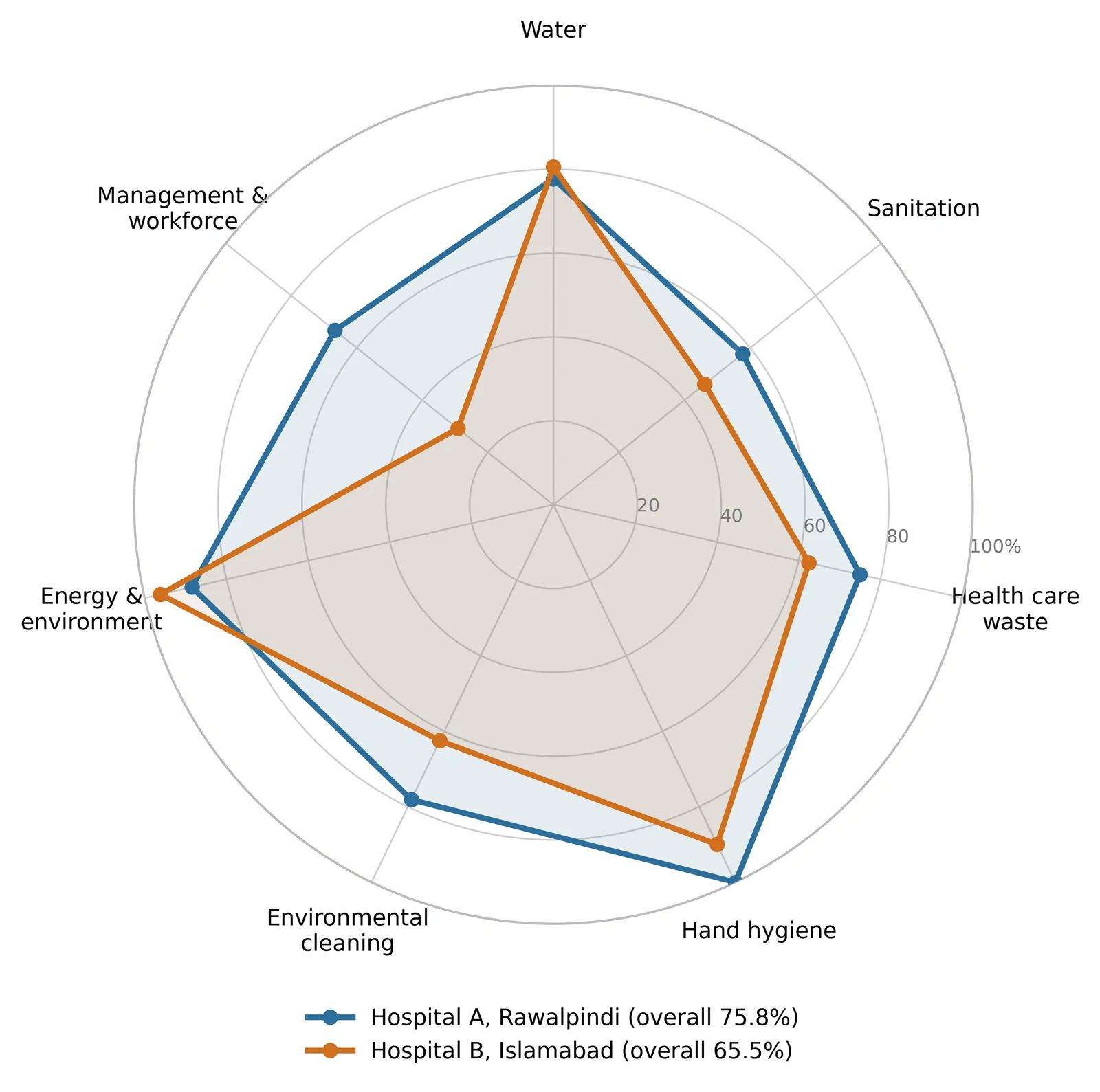
Domain-wise WASH FIT performance in the two hospitals WASH FIT: Water and Sanitation for Health Facility Improvement Tool

Hospital A achieved 147 of a maximum 194 points (75.8%), and Hospital B achieved 127 of 194 points (65.5%), a difference of 10.3 percentage points. Both facilities therefore demonstrated moderate overall water, sanitation and hygiene (WASH) performance, with Hospital A performing better. The distribution of individual scores underlying these totals differed appreciably between the facilities. Hospital A met 69 of 97 indicators fully (71.1%), partially met 9 (9.3%), and failed 19 (19.6%). Hospital B met 57 indicators fully (58.8%), partially met 13 (13.4%), and failed 27 (27.8%). The difference between the two facilities is thus driven less by widespread partial compliance than by a larger number of indicators left entirely unmet in Hospital B. Domain-level performance is presented in Table 1 and illustrated in Figure 1. Hospital A scored higher in five of the seven domains. Hospital B scored higher in water, 29/36 (80.6%) versus 28/36 (77.8%), and in energy and environment, 25/26 (96.2%) versus 23/26 (88.5%).

**Table 1:** Domain-wise WASH FIT scores and JMP service levels pp = percentage points. Service levels follow JMP core-indicator criteria and are not defined for the energy and management domains. WASH FIT: Water and Sanitation for Health Facility Improvement Tool; JMP: Joint Monitoring Programme

Domain	n	Hospital A score	Hospital A %	Hospital B score	Hospital B %	Diff (pp)	Level A	Level B
Water	18	28/36	77.8	29/36	80.6	−2.8	Basic	Basic
Sanitation	13	15/26	57.7	12/26	46.2	+11.5	Basic	Limited
Health care waste	20	30/40	75.0	25/40	62.5	+12.5	Limited	Limited
Hand hygiene	5	10/10	100.0	9/10	90.0	+10.0	Basic	Basic
Environmental cleaning	16	25/32	78.1	20/32	62.5	+15.6	Basic	Limited
Energy and environment	13	23/26	88.5	25/26	96.2	−7.7	—	—
Management and workforce	12	16/24	66.7	7/24	29.2	+37.5	—	—
Overall	97	147/194	75.8	127/194	65.5	+10.3		

**Figure 2:**
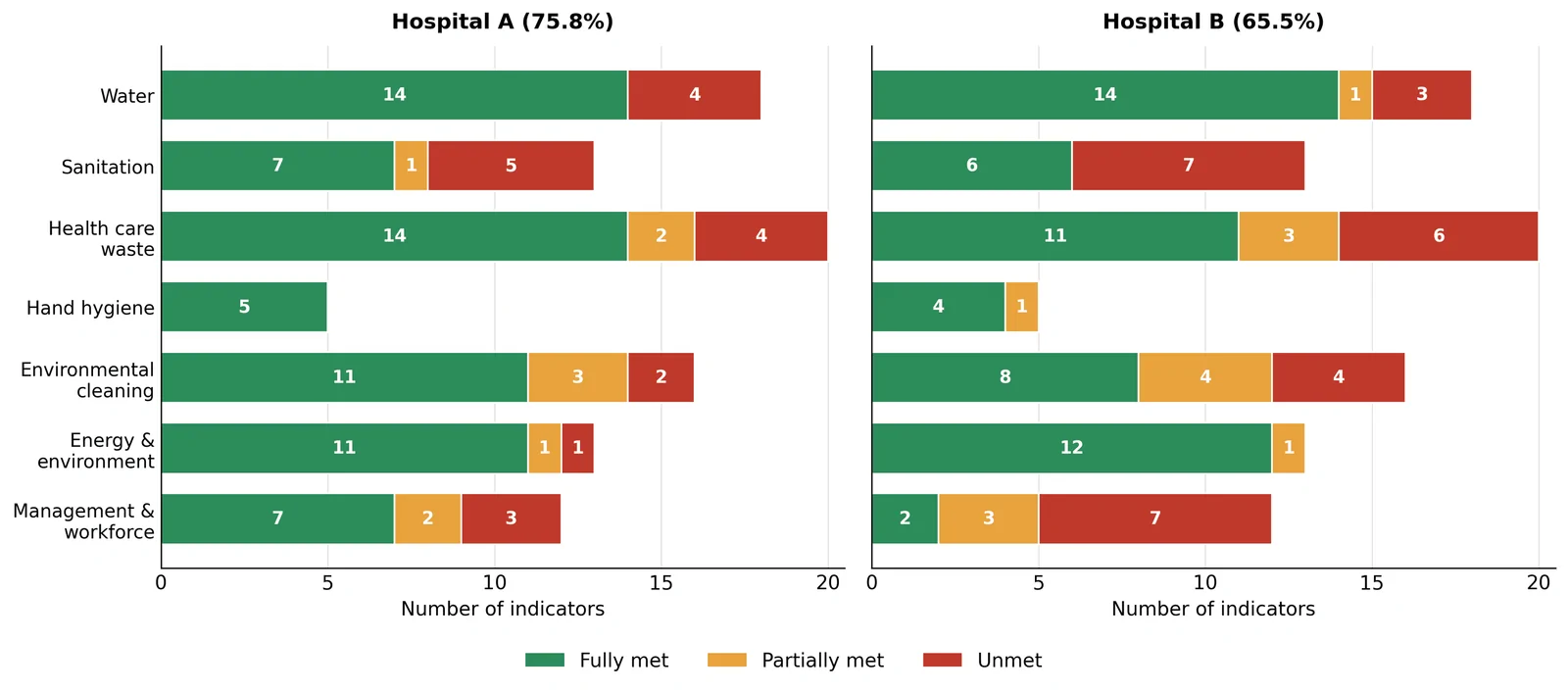
Distribution of fully met, partially met, and unmet WASH FIT indicators by domain in the two hospitals WASH FIT: Water and Sanitation for Health Facility Improvement Tool

Hospital A met the criteria for a basic service in four of the five domains for which JMP service ladders are defined, compared with two of five in Hospital B. Hospital B was classified at a limited-service level for sanitation and environmental cleaning. Health care waste was classified as limited in both facilities. The composition of indicator scores underlying these domain classifications is shown in Figure 2. In both facilities, the unmet indicators were concentrated in sanitation, health care waste, and management and workforce, whereas water and hand hygiene were almost entirely fully met; the larger unmet burden in Hospital B was most pronounced in the management and workforce domain.

Domain-level findings

Water

Hospital A scored 28/36 (77.8%) and Hospital B 29/36 (80.6%); both were classified at a basic service level. Both facilities had an improved on-premises water supply, functional piped distribution, water available on all days of the week, sufficient quantity, secondary supply arrangements, and drinking water meeting facility requirements. Rainwater harvesting and regulated treatment of piped water with documented safe water management were unmet in both. Emergency water storage and water reduction strategies were unmet in Hospital A and fully met in Hospital B. Shower provision for inpatients was partially met, and a private, lockable washing space for women in the labor and delivery area was unmet in Hospital B; both were fully met in Hospital A.

Sanitation

Hospital A scored 15/26 (57.7%) and was classified at a basic service level. Improved toilets were available in sufficient number, with a separate staff toilet, sex-separated provision with privacy, a toilet meeting menstrual hygiene management needs, and an accessible toilet for users with limited mobility. Hospital B scored 12/26 (46.2%) and was classified at a limited-service level, with no toilet meeting menstrual hygiene management needs. Availability and usability of all patient toilets were partially met in Hospital A and fully met in Hospital B; leak-free connection to the public sewer was fully met in Hospital A and unmet in Hospital B. Five indicators were unmet in both facilities: containment of faecal sludge for off-site treatment, a functioning wastewater treatment plant with operational records, stormwater and greywater drainage, capture and reuse of stormwater or greywater, and safe greywater capture from sinks and laundry.

Health Care Waste

Hospital A scored 30/40 (75.0%) and Hospital B 25/40 (62.5%); both were classified at a limited-service level. Both facilities had functional waste collection containers, segregation reminders, a dedicated storage area, appropriate storage duration for infectious waste, safe and regular off-site collection and treatment, dedicated handling of anatomical and pharmaceutical waste, a trained waste management focal person, occupational protection for waste handlers, and emergency surge preparedness. Correct segregation at all waste generation points was partially met in both, and recycling of non-hazardous waste was unmet in both. Four indicators scored higher in Hospital A: protective equipment and hand hygiene resources for waste handling staff, and training and reminders on their rational use (both fully met in Hospital A, partially met in Hospital B); and facility-wide waste minimization strategies and flood-resistant construction of waste pits (met and partially met respectively in Hospital A, both unmet in Hospital B).

Hand Hygiene

Hospital A scored 10/10 (100%) and Hospital B 9/10 (90%); both were classified at a basic service level. Hand hygiene supplies were consistently available, compliance was monitored, and staff had received training in both facilities. The facilities differed on one indicator: functioning hand hygiene stations at all points of care, including the delivery room, which was partially met in Hospital B.

Environmental Cleaning

Hospital A scored 25/32 (78.1%) and was classified at a basic service level; Hospital B scored 20/32 (62.5%) and was classified at a limited-service level. Both facilities had sufficient cleaning staff, appropriate supplies and equipment, defined cleaning schedules, and routine cleaning of surfaces. A displayed and implemented cleaning policy or protocol and occupational safety policies for cleaners and waste technicians were fully met in Hospital A and unmet in Hospital B. Training of cleaning staff and provision of a dedicated area for storage and preparation of cleaning supplies were both partially met in Hospital B. Functional laundry facilities able to meet demand and hot-water laundry reprocessing at 70-80 °C were unmet in both facilities.

Energy and Environment

Hospital A scored 23/26 (88.5%) and Hospital B 25/26 (96.2%). Both facilities had a reliable primary electricity supply, functional backup generation, adequate lighting in clinical and delivery areas, sufficient ventilation, and functional temperature control. Insecticide-treated bed nets were partially provided in both. The facilities differed on one indicator: sustainable life-cycle procurement, which was met in Hospital B and unmet in Hospital A.

Management and Workforce

Hospital A scored 16/24 (66.7%) and Hospital B 7/24 (29.2%), a difference of 37.5 percentage points. Hospital A fully met requirements for a functional quality improvement, IPC or WASH FIT team; written job descriptions for auxiliary, cleaning, and waste-handling staff; induction training in WASH and IPC for new auxiliary staff; annual staff performance appraisal; and an operational patient safety policy. In Hospital B, job descriptions and induction training were unmet, and the remaining three were partially met. Representation of user and staff groups and a displayed facility management structure diagram were partially met in Hospital A and unmet in Hospital B. Three indicators were unmet in both facilities: a dedicated WASH focal person or engineer working to an approved programme of work, a facility-wide environmental sustainability policy, and a budgeted, regularly updated emergency preparedness and response plan with staff drills.

Performance by indicator type

WASH FIT classifies indicators as essential or advanced, and separately identifies those forming the JMP core monitoring set and those relevant to climate resilience. Disaggregating performance by indicator type revealed a consistent pattern not visible in the domain scores (Table 2).

**Table 2:** Performance by indicator classification Classes are not mutually exclusive and do not sum to the analytical set; a minority of indicators are classified by service configuration or facility type rather than by priority level.

Indicator class	n	A score	A %	B score	B %	Diff (pp)
Essential	41	76/82	92.7	67/82	81.7	+11.0
Advanced	41	50/82	61.0	41/82	50.0	+11.0
JMP core set	14	26/28	92.9	21/28	75.0	+17.9
Climate resilience	13	5/26	19.2	10/26	38.5	−19.2

Both hospitals scored higher on essential than on advanced indicators: 76/82 (92.7%) versus 50/82 (61.0%) in Hospital A and 67/82 (81.7%) versus 41/82 (50.0%) in Hospital B, a decrement of 31.7 percentage points in each facility. Performance on the JMP core monitoring set was 26/28 (92.9%) in Hospital A and 21/28 (75.0%) in Hospital B. Climate resilience scored 5/26 (19.2%) in Hospital A and 10/26 (38.5%) in Hospital B. In Hospital A, it fell below every domain score recorded in either facility; in Hospital B, only management and workforce, 7/24 (29.2%), scored lower; Hospital B scored higher than Hospital A on this dimension. Seven of the 13 climate-related indicators were unmet in both facilities: rainwater harvesting, stormwater and greywater drainage, capture and reuse of stormwater or greywater, safe greywater capture from sinks and laundry, recycling of non-hazardous waste, an environmental sustainability policy, and a budgeted emergency preparedness plan.

Concordance and shared gaps

Of the 97 comparable indicators, 72 (74.2%) received an identical score in both hospitals. Of the 25 discordant indicators, 20 favored Hospital A, and 5 favored Hospital B. The two facilities therefore share a common performance profile, with differences concentrated in a minority of indicators rather than distributed across the assessment. Sixteen indicators (16.5%) scored zero in both facilities, as shown in Table 3. One is classified as essential and eleven as advanced; the remaining four carry no priority designation, three being specific to on-site waste treatment and one classified only for climate relevance. Seven relate to climate resilience. Their clustering in wastewater and stormwater management, waste minimization, laundry infrastructure, and institutional planning indicates deficits in provision that is procured, financed, or regulated above the level of the individual facility.

**Table 3:** Indicators unmet in both facilities Three indicators (HCWM_10, HCWM_11, HCWM_15) apply only to facilities operating on-site waste treatment and are excluded in the sensitivity analysis; thirteen indicators are unmet in both facilities when these are treated as not applicable. WASH: Water, sanitation and hygiene

Domain	Indicator	Requirement	Classification
Water	W_9	Functional rainwater harvesting with safe storage	Advanced, climate
Water	W_13	Regulated treatment of piped water with safe water management	Advanced
Sanitation	S_8	Faecal sludge fully contained for treatment	Advanced
Sanitation	S_10a	Functional wastewater treatment plant with operational records	Advanced
Sanitation	S_11	Stormwater and greywater drainage system	Essential, climate
Sanitation	S_12	Capture and reuse of stormwater or greywater	Advanced, climate
Sanitation	S_13	Safe greywater capture from sinks and laundry	Advanced, climate
Health care waste	HCWM_7	Segregation and recycling of non-hazardous waste	Climate
Health care waste	HCWM_10	On-site waste treatment technology	Configuration-specific
Health care waste	HCWM_11	Energy or fuel supply for on-site treatment	Configuration-specific
Health care waste	HCWM_15	Dedicated ash pits for incineration residues	Configuration-specific
Environmental cleaning	EC_13	Laundry facilities able to meet demand	Advanced
Environmental cleaning	EC_14	Hot-water laundry reprocessing at 70-80 °C	Advanced
Management	M_2	Dedicated WASH focal person with approved programme of work	Advanced
Management	M_11	Facility-wide environmental sustainability policy	Advanced, climate
Management	M_12	Budgeted emergency preparedness and response plan	Advanced, climate

Sensitivity analysis

Three health care waste indicators in the analytical set apply only to facilities operating on-site waste treatment: on-site treatment technology, fuel supply for that technology, and ash pits for incineration residues. Both hospitals use off-site treatment and scored the maximum on the corresponding off-site indicator, so these three arguably do not apply to either facility. Treating them as not applicable in both hospitals raises the health care waste score from 30/40 (75.0%) to 30/34 (88.2%) in Hospital A and from 25/40 (62.5%) to 25/34 (73.5%) in Hospital B, and raises overall performance from 147/194 (75.8%) to 147/188 (78.2%) and from 127/194 (65.5%) to 127/188 (67.6%) respectively. The relative position of the facilities is unchanged, and no service level classification is affected.

## Discussion

This study provides a comparative assessment of water, sanitation and hygiene (WASH) services in two large hospitals using the WHO Water and Sanitation for Health Facility Improvement Tool (WASH FIT) framework, demonstrating moderate overall performance with persistent domain-specific gaps. These findings are consistent with recent global and regional evidence indicating that, despite increasing recognition of WASH as a core component of quality care, substantial deficiencies remain across healthcare facilities, particularly in LMICs.

The overall performance observed in both hospitals (147/194, 75.8% in Hospital A and 127/194, 65.5% in Hospital B) reflects moderate WASH service levels. Similar patterns have been reported in multi-country analyses, where a significant proportion of facilities fail to achieve comprehensive WASH standards across all domains [11]. Likewise, a multicentre assessment of 14 public hospitals in Kenya reported overall WASH performance ranging from 47% to 71% (median 61%), indicating that variability in WASH performance across healthcare facilities is common in low- and middle-income settings [12]. The inter-facility differences observed in the present study are therefore consistent with previous evidence and appear to be associated with variations in governance, resource allocation, and institutional capacity rather than infrastructure alone [13].

Both hospitals achieved strong performance in water and hand hygiene domains, consistent with existing literature showing that these are typically the most developed components of WASH systems in healthcare settings [14]. Reliable water supply and availability of hand hygiene facilities are often prioritized due to their direct role in infection prevention [15]. Evidence indicates that improvements in hand hygiene alone significantly reduce healthcare-associated infections (HAIs), making it one of the most cost-effective interventions in clinical settings [16].

Despite acceptable performance in some domains, health care waste management remained at a limited-service level in both hospitals, and sanitation in Hospital B. This aligns with global findings that sanitation is frequently the weakest domain in WASH assessments, with up to 60% of facilities lacking adequate sanitation services [17]. Poor sanitation infrastructure, absence of wastewater treatment, and inadequate waste management systems increase the risk of environmental contamination and occupational exposure, particularly among support staff [18].

The difference observed between the two hospitals in environmental cleaning is notable. Evidence suggests that environmental hygiene is a very important yet often under-addressed component of infection prevention systems [19]. Inadequate cleaning protocols, lack of standardized procedures, and insufficient training contribute to persistent contamination risks in healthcare environments [20]. The lower performance in Hospital B may reflect these systemic issues, particularly in the absence of formalized operational policies.

One of the most significant findings of this study is the disparity in the management and workforce domain, particularly the markedly lower performance in Hospital B. This is consistent with emerging evidence that governance structures, leadership engagement, and institutional accountability are among the strongest correlates of WASH performance [21].

Governance deficiencies and possible interventions

These governance deficiencies have direct practical consequences, since management indicators underpin the sustainability of gains made in the more visible service domains. Several of the shortcomings identified are amenable to low-cost, administratively driven interventions that do not depend on major capital investment.

First, neither facility had designated a dedicated WASH focal person or engineer working to an approved programme of work; appointing such a focal point, with a defined budget line and clear reporting to senior leadership, provides the accountability structure through which the remaining gaps can be addressed.

Second, the weaker performance of Hospital B was driven substantially by the absence of written job descriptions and structured induction training for auxiliary, cleaning, and waste-handling staff; incorporating explicit WASH and infection prevention responsibilities into staff job descriptions, coupled with a short standardized induction module and periodic refresher training, is an inexpensive measure that directly strengthens frontline practice.

Third, activating a functional quality-improvement or WASH FIT committee with defined membership and scheduled review cycles would institutionalize the continuous-improvement process that the tool is designed to support, and would allow annual performance appraisal already partially in place in Hospital B, to be linked explicitly to hand hygiene and IPC indicators.

Finally, both facilities lacked a budgeted emergency preparedness and response plan with staff drills, and an environmental sustainability policy; formulating these as written, costed, and regularly reviewed documents would close two of the indicators unmet across both sites and improve resilience to climate-related and other disruptions. Collectively, these measures are governance and workforce interventions rather than infrastructural ones, and are therefore realistically achievable within existing resources, reinforcing the study's central observation that the largest observed differences in WASH performance in these facilities lay in managerial rather than structural.

The application of WASH FIT in this study enabled a comprehensive, domain-specific assessment and facilitated identification of actionable gaps. WASH FIT is increasingly recognized as a practical, risk-based tool that supports continuous quality improvement in healthcare facilities by integrating environmental health into broader health system strengthening efforts [22]. The findings of this study have important implications for health systems in resource-limited settings. Persistent gaps in sanitation, waste management, and governance highlight the need for integrated, system-level interventions rather than isolated infrastructural improvements. Strengthening WASH services requires coordinated efforts involving policy, financing, workforce development, and monitoring systems.

Limitations

This study has several limitations that should be considered when interpreting the findings. First, the assessment was confined to two hospitals within a single healthcare network. Because both facilities share common ownership, governance arrangements, procurement systems, and staffing and training policies, they may exhibit organizational similarities that are not representative of hospitals in other networks or sectors, and the findings should not be generalized to facilities operating under different management structures. This shared organizational context is, however, also a strength for the internal comparison: it holds constant many system-level factors, so that the differences observed between the two facilities are less likely to be confounded by network-level variation. The trade-off is therefore one of narrower external validity in exchange for greater internal comparability.

Second, the cross-sectional design captures WASH conditions at a single point in time and cannot detect temporal variation or change following intervention. WASH performance fluctuates with operational, seasonal, and resource-related factors.

Third, the assessment relied on structured observation and review of facility records without microbiological sampling or quantitative environmental measurement. Indicators concerning drinking water quality, environmental cleanliness, and laundry disinfection were therefore evaluated against documented practice and observable provision rather than verified performance, and may overstate actual service quality.

Fourth, the assignment of ordinal scores through structured observation inevitably involves a degree of assessor judgement, particularly for indicators partially met. To mitigate this, each facility was assessed independently by two separate teams, discrepancies were reconciled against the supporting observational and documentary evidence, and final scores were adjudicated by an author not affiliated with either participating facility. Agreement between the two teams' independent scores prior to consensus was high (Cohen's κ = 0.94), indicating reasonably consistent scoring across assessors. Nevertheless, consensus-based scoring, while reducing single-observer error, does not eliminate the possibility of shared interpretive bias between teams applying the same tool, and some residual subjectivity cannot be excluded; findings for indicators dependent on observation and documentation should be interpreted with this in mind.

Fifth, several WASH FIT indicators apply only to facilities with particular service configurations. Three health care waste indicators relevant only to on-site treatment were scored as unmet in facilities that use off-site treatment, and the sensitivity analysis presented above shows that this depressed the health care waste and overall scores in both hospitals. Reported domain percentages should therefore be interpreted as conservative estimates, and caution is warranted when comparing them with studies that have handled configuration-specific indicators differently. Finally, WASH FIT percentage scores and JMP service levels measure different things and are not interchangeable. A domain may record a high percentage while remaining at a limited-service level because a single core indicator is unmet, as observed here for health care waste. Percentage scores describe overall provision across a domain, whereas service levels reflect performance against defined minimum standards, and both are reported for this reason.

## Conclusions

This comparative assessment found that both hospitals demonstrated moderate overall WASH performance, with particular strengths in water supply and hand hygiene and persistent gaps in sanitation, health care waste management, and institutional governance. The largest difference between the two facilities lay in the management and workforce domain rather than in physical infrastructure, indicating that, in these two hospitals, governance and workforce factors rather than structural provision accounted for most of the observed variation in WASH performance.

On this basis, we suggest that strengthening WASH services in resource-limited healthcare facilities is likely to require integrated, system-level strategies addressing governance and workforce capacity alongside infrastructure, rather than infrastructural improvement alone.
